# Perspective of Melatonin-Mediated Stress Resilience and Cu Remediation Efficiency of *Brassica juncea* in Cu-Contaminated Soils

**DOI:** 10.3389/fpls.2022.910714

**Published:** 2022-07-18

**Authors:** Anayat Rasool Mir, Pravej Alam, Shamsul Hayat

**Affiliations:** ^1^Plant Physiology Section, Department of Botany, Faculty of Life Sciences, Aligarh Muslim University, Aligarh, India; ^2^Department of Biology, College of Science and Humanities in Al-Kharj, Prince Sattam Bin Abdulaziz University, Al-Kharj, Saudi Arabia

**Keywords:** antioxidant, bioaccumulation, chloroplast, oxidative stress, phytoremediation

## Abstract

The present study evaluated the influence of melatonin (MEL) on copper toxicity in terms of morphophysiological, microscopic, histochemical, and stress resilience responses in *Brassica juncea*. Different levels of Cu (0, 30, and 60 mg kg^–1^) were given in air-dried soil, and 25 days after sowing (DAS), plants were sprayed with 30, 40, or 50 μM of MEL. The results demonstrated that under Cu stress, a significant amount of Cu accumulated in plant tissues, particularly in roots than in upper ground tissues, thereby suppressing the overall growth as evidenced by decrease in tolerance index and photosynthesis and increase in oxidative stress biomarkers (reactive oxygen species, malondialdehyde, and electrolyte leakage content) and cell death. Interestingly, the follow-up treatment of MEL, mainly 40 μM, efficiently improved the physio-biochemical and growth parameters, sugar accumulation, and metabolism. The potential of MEL in modulating Cu stress is attributed to its involvement in enriching the level of nutrient and improving chloroplast and stomatal organization besides lowering oxidative stress *via* enhanced levels of antioxidants. MEL improved the Cu reclamation potential in plants by enhancing Cu uptake and its translocation to aerial tissues. Principal component analysis showed that most of the morphophysiological and growth attributes were positively linked with MEL and negatively related to Cu levels, whereas all the stress-enhancing attributes showed a strong relationship with excessive Cu levels in soils. The present study suggested that MEL has the potential to improve growth and photosynthesis resulting in improved stress resilience under Cu stress along with increased remediation capability of mustard for remediation of Cu-contaminated soils.

## Introduction

In the natural environment, plants are generally exposed to different stresses that suppress plant growth and productivity and consequently affect global food security. In this perspective, heavy metal stress is one of the critical problems in agricultural soils. The non-biodegradable, persistent behavior of heavy metals and their tendency to accumulate in soils have turned out to be a major concern in agricultural productivity. Despite copper (Cu) being an essential mineral for plant growth, its excessive levels are highly phytotoxic to plants (Saez et al., 2015). Cu concentration in agricultural soil has exponentially increased because of ever-increasing anthropogenic activities such as excessive use of Cu-based pesticides, nematicides, and fungicides, and use of sewage wastes (Brun et al., 2001). Such excessive Cu levels pose a severe threat to the environment and agricultural productivity and ultimately affect human health (Mir et al., 2021a). Cu imposes phytotoxicity by morphophysiological and anatomical alterations, metabolic and biochemical disturbances that hamper plant growth. Moreover, excessive levels of Cu inhibit photosynthesis, nutrient assimilation, and sugar accumulation, and decreases membrane stability by triggering the production of reactive oxygen species (ROS) and lipid peroxidases (LPOs) (Saleem et al., 2020). Hence, it is desirable to develop sustainable techniques for effective reclamation of Cu-contaminated soil. Among various methods, phytoremediation is a conventional eco-friendly technology in which plants with higher affinity for heavy metals are grown to remediate toxic soil pollutants (Yan et al., 2020). However, toxic heavy metal’s availability in the contaminated soil needs to be determined besides measuring the capability of plants to maintain growth under metal-polluted conditions. For instance, if the toxicant bypasses the plant tolerance limit, it may induce toxicity and inhibit plant growth. This will ultimately decrease the remediation efficiency of plants. For this reason, a systematic experimental consideration of Cu-imposed modulations in morpho-physiological attributes, nutrient levels, antioxidant defense system, and oxidative biomarkers must be performed to determine the plant capability to remove the metal from contaminated soil and to retain in the shoot.

For this reason, a systematic experimental consideration of Cu-imposed modulations in morpho-physiological attributes, nutrient levels, antioxidant defense system and oxidative biomarkers must be performed to determine the plant capability to remove the metal from soil and to retain in the shoot. Additionally, it is essential to improve the growth performance as well as remediation potential of plants grown on Cu-polluted soils. Considering this, numerous studies reported pathways for neutralizing the phytotoxic effects of Cu stress and improving Cu tolerance in plants’ by exogenous application of PGRs (Ben Massoud et al., 2019).

Melatonin (MEL) and its intermediates are among the best antioxidant molecules known for their hydrophilic and hydrophobic properties. MEL easily flows through the cell membrane and allocates among aqueous cell compartments such as mitochondria, nucleus, and cytosol (Galano et al., 2013). It regulates various signaling pathways in plants under multiple stress conditions, and it primarily functions as an antioxidant and a growth promoter in plants (Arnao and Hernández-Ruiz, 2015; Mir et al., 2020). Numerous studies have explored the scavenging role of MEL in improving stress tolerance in plants. For instance, MEL induces tolerance in crops under heavy metals (Hoque et al., 2021), drought (Imran et al., 2021), high temperature (Jahan et al., 2019), cold stress (Turk et al., 2014), and salinity (Farouk and Al-Huqail, 2022) conditions. During stress conditions, MEL enhances a wide range of adaptive responses such as improvement of photosynthesis and gas exchange attributes (chlorophyll content, stomatal conductance, photosynthetic rate, and transpiration rate), enrichment of nutrient levels, improvement of sugar metabolism and hormonal regulation, enhancement of secretion of organic acid anions and phenolic compounds, and improvement of ROS scavenging *via* improved antioxidant defense system, thus reducing lipid peroxidation and oxidative burst in plant cells (Farouk and Al-Amri, 2019; Farouk and Al-Huqail, 2022). However, limited information is available on connection with the mitigating efficiency of MEL in countering the negative impact of excess Cu stress. A preliminary study includes seed hydropriming with MEL to improve germination percentage under Cu^2+^ stress conditions (Posmyk et al., 2008), improved Cu tolerance and antioxidant activities in pea plants (Tan et al., 2007), and enhanced Cu sequestration and ROS scavenging in cucumber (Cao et al., 2019). However, the mechanisms of melatonin-mediated tolerance in Cu^2+^ stress remain elusive.

*Brassica juncea* is an important oilseed crop and accounts for nearly 30% of overall oilseed stock in India. It has been reported that its oil production and yield have declined notably on hazardous metal-polluted soils (Mir et al., 2021). Furthermore, its production in India is comparatively insufficient vis-à-vis other mustard-producing countries. Hence, this oilseed crop is chosen for the current study. To monitor the protective function of MEL in copper toxicity in terms of metal accumulation, Cu phytoremediation, and stress resilience in *Brassica juncea*; the present set of experiment was conducted, in which we have investigated the interactive role of different MEL levels on growth, physio-biochemical attributes, ultrastructural and morphological alterations, and its stress ameliorating ability under Cu stress. This study brings forth novel insights by incorporating and evaluating ultrastructural modifications at the level of chloroplast anatomy and stomatal morphology by electron microscopy, histochemical and microscopic analyses of cell death, and ROS localization analysis by confocal laser microscopic and histochemical analyses of stress biomarkers, which has improved the perception of MEL ability to mitigate Cu toxicity in plants.

## Materials and Methods

### Hormone Preparation

Melatonin was purchased from Sigma-Aldrich. A stock solution (100 μM) was prepared by dissolving a required quantity of melatonin in 5 ml of ethanol and then diluted with DDW to make a 100-ml solution. The required concentrations of MEL (10, 20, 30, 40, and 50 μM) were formulated by diluting the stock solution in DDW.

### Biological Material

Certified seeds of *Brassica juncea* var. Varuna were purchased from Seed Bhandar (Aligarh, India). Healthy and regular-sized seeds were selected and surface-sterilized with 0.1% mercuric chloride solution for 10–20 min followed by repeated washing with DDW.

### Experimental Design

The experiment was conducted in a randomized block design with 60 clay closed pots stuffed with 4 kg of soil and farmyard manure kept in a 3:1 ratio and were distributed in 12 sets with 5 pots (replicates) to represent one treatment, under net house conditions, in the Department of Botany, Aligarh Muslim University, India. The physiochemical characteristics of soil are determined and are given in Supplementary Table 1. Before application of Cu (CuSO_4_), the soil was tilled and required amounts of Cu (30, 60, and 90 mg kg^–1^, dissolved in 250 ml of DDW) were poured into the soil according to field capacity. Furthermore, Cu content in the soil before and after exogenous application was determined. Three plants in each pot were maintained. In the 45-day stage, upper canopy leaf samples (upper 3rd leaf, five samples from each treatment) were taken to evaluate vegetative growth characteristics in terms of tolerance index, gas exchange parameters, stress biomarkers (ROS, lipid peroxidation, and membrane stability), biochemical parameters (NR, CA, rubisco activity, and carbohydrate metabolic enzymes), and nutrient status, and microscopic analysis of chloroplast, stomata, cell death, and ROS localization. The scheme of treatment was as follows:

Set I: Control (plants were sprayed DDW in the absence of Cu).
Set II: Cu at a rate of 30 mg kg^–1^ was added to the soil.
Set III: Cu at a rate of 60 mg kg^–1^ was added to the soil.
Set IV: MEL at a rate of 30 μM was applied to the foliage (25–29 DAS).
Set V: MEL at a rate of 40 μM was applied to the foliage (25–29 DAS).
Set VI: MEL at a rate of 50 μM was applied to the foliage (25–29 DAS).
Set VII: Cu at a rate of 30 mg kg^–1^ was added to the soil, and plants were sprayed with 30 μM of MEL (25–29 DAS).
Set VIII: Cu at a rate of 30 mg kg^–1^ was added to the soil, and plants were sprayed with 40 μM of MEL (25–29 DAS).
Set IX: Cu at a rate of 30 mg kg^–1^ was added to the soil, and the plants were sprayed with 50 μM of MEL (25–29 DAS).
Set X: Cu at a rate of 60 mg kg^–1^ was added to the soil, and the plants were sprayed with 30 μM of MEL (25–29 DAS).
Set XI: Cu at a rate of 60 mg kg^–1^ was added to the soil, and the plants were sprayed with 40 μM of MEL (25–29 DAS).
Set XII: Cu at a rate of 60 mg kg^–1^ was added to the soil, and the plants were sprayed with 50 μM of MEL (25–29 DAS).

### Stress Tolerance Index

Stress tolerance indices were assessed for plant height (PH) and vegetative growth (VG), which were cited as TiPH and TiVG, respectively, applying the following equations:


$$\text{TiPH}=\frac{\mathrm{Height}\,\mathrm{of}\,\mathrm{Treated}\,\mathrm{plant}}{\mathrm{Height}\,\mathrm{of}\,\mathrm{Control}\,\mathrm{plant}}*100$$

$$\text{TiVG}=\frac{\mathrm{FW}\,\mathrm{of}\,\mathrm{Treated}\,\mathrm{plant}}{\mathrm{FW}\,\mathrm{of}\,\mathrm{Control}\,\mathrm{plant}}*100$$

### Relative Water Content

The relative water content (RWC) in the leaves was determined by the method described by Hayat et al. (2007). Fully expanded leaves were plucked and sliced into circular discs (2 cm width) and weighed instantly. The discs were placed in a shaded Petri plate filled with DDW for 24 h. Thereafter, the discs were carefully picked to estimate their turgor weight. The leaf samples were oven-dried for 48 h at 70°C to estimate their dry weight.

RWC was calculated by the following formula:


$$\mathrm{RWC}\%=\frac{\mathrm{FW}-\mathrm{DW}}{\mathrm{TW}-\mathrm{DW}}*100$$

where FW is fresh weight, DW is dry weight, and TW is turgid weight.

### Leaf SPAD Value

Total chlorophyll content was evaluated in a third fully developed leaf at noon with a SPAD chlorophyll meter (SPAD–502; Konica Minolta Sensing, Inc., Japan).

### Gas Exchange Parameters

Net photosynthetic rate (*P*_*N*_), stomatal conductance (*gs*), internal CO_2_ concentration (C_*i*_), and transpiration rate (E) were analyzed in upper fully developed leaves with an infrared gas analyzer (IRGA) photosynthetic system (LI-COR 6400; LICOR, Lincoln, Nebraska, FL, United States). On a sunny day, estimations were held at 11:00 a.m. and 12:00 noon. The IRGA was calibrated to adjust the atmospheric temperature (22 ± 1°C), relative humidity (60 ± 3%), photosynthetically active radiation (1,016 ± 6 μmol m^–2^ s^–1^), and atmospheric CO_2_ (600 μmol mol^–1^) (Mir et al., 2021a).

### Chlorophyll Fluorescence

Chlorophyll fluorescence was determined using a Junior PAM chlorophyll fluorometer (Heinz Walz, Germany). Actual PSII efficiency, maximum PSII efficiency, electron transport rate, and photochemical and non-photochemical quenching were evaluated like in our earlier studies (Mir et al., 2021).

### Rubisco Activity

The activity of Rubisco (E.C.4.1.1.39) was determined with the standardized method demonstrated by Usuda (1985). Freshly collected leaves were pulverized in a solution containing MgCl_2_, Tris–HCl, EDTA, and DTT, and centrifuged (HERMLE LABORTECHNIK, Z327K, Germany) at 10,000 × g for 10 min. The supernatant was collected to which 100 mM Tris–HCl (pH 8), 0.2 mM EDTA, 4 mM ATP, 0.2 mM NADH, 10 mM MgCl_2_, 40 mM NaHCO_3_, 5 mM DTT, and 1 U of 3-kinase were added. Rubisco activity was measured by adding 0.2 mM ribulose 1,5-bisphosphate (RuBP).

### Total Soluble and Reducing Sugars

Dried powder (50 mg) of leaf samples was crushed in 80% ethanol and homogenated at 10,000 × g for 1 h. The supernatant was collected and used to determine the reducing and total soluble sugar contents (Mir et al., 2021a).

Total soluble sugar and reducing sugar were estimated following the method described in our earlier studies (Mir et al., 2020). Absorbance was measured at 485 nm (Spekol 1500 UV VIS spectrophotometer) for total soluble sugar and at 560 nm for reducing sugar, and sugar content was calculated using a standard solution of D-glucose.

### Starch Content

After ethanolic extraction, the residue was hydrolyzed with perchloric acid (52%). One mL of extract was collected to which 10 ml of an ice-cold anthrone reagent and 4 ml of DDW were added. The solution was perturbed briskly and readily heated on a water bath. The absorbance was evaluated at 630 nm using a spectrophotometer (Spekol 1500 UV VIS spectrophotometer).

### Glucose, Fructose, and Sucrose Content

The glucose, fructose, and sucrose content in the leaves was determined with the method described by Siddiqui et al. (2020).

### Hexokinase

Leaf hexokinase (HXK) (E.C.2.7.1.1) was extracted with the method of Whittaker et al. (2001), followed by that of Bergmeyer et al. (1983) to estimate HXK activity. A leaf sample (.5 g) was crushed in liquid nitrogen, to which a 50-mM triethanolamine buffer and polyvinylpyrrolidone (PVP) were added. The sample mixture was transferred to a pre-chilled buffer solution comprising a 19-mM adenosine 5′-triphosphate solution, a 555-mM D-glucose solution, 14 mM β-nicotinamide adenine dinucleotide phosphate, a 100-mM magnesium chloride solution, and a glucose-6-phosphate dehydrogenase solution. The mixture was mixed properly and equilibrated at 25°C, to which 0.05 ml of an HXK enzyme solution was added. Optical density was read at 340 nm for 5 min.

### Fumarase and Succinate Dehydrogenase

Fumarase (FH) (E.C.4.2.1.2) and succinate dehydrogenase (SDH) (E.C.1.3.5.1) activity in leaves was estimated as described by Siddiqui et al. (2020).

### Nitrate Reductase and Carbonic Anhydrase

Leaf nitrate reductase (NR) (E.C.1.7.99.4) and carbonic anhydrase (CA) (E.C.4.2.1.1) activities were assessed with the method described by Jaworski (1971) and Dwivedi and Randhawa (1974), respectively. The detailed methodologies were described in our earlier study (Mir et al., 2020).

### Superoxide Anion (O_2_^–^) and Hydrogen Peroxide (H_2_O_2_) Level

Superoxide anion content and H_2_O_2_ in the leaves were estimated as per our previous studies (Mir et al., 2020).

### Lipid Peroxidation Content

Lipid peroxidation (MDA) was evaluated by calculating MDA content in the leaves. The detailed procedure is described in our earlier study (Mir et al., 2020).

### Visualization of O_2_^–^ and H_2_O_2_ Level

Superoxide anion (O_2_^–^) and hydrogen peroxide (H_2_O_2_) localization were determined with the method demonstrated by Kumar et al. (2014).

### Lipid Peroxide Localization

Localization of lipid peroxidation was performed with the method demonstrated by Awasthi et al. (2018). Freshly collected leaf and root samples were washed and immersed in a shiff reagent solution. Afterward, the samples were rinsed with a sulfite solution to retain the color of stain. Images were captured using a digital camera.

### Proline and Glutathione Content

Proline content in the leaves was estimated with the method of Bates et al. (1973) and GSH with the method of Sedlak and Lindsay (1968).

### Antioxidant Enzyme Activity

Leaves of *Brassica juncea* were crushed in the extraction buffer as described earlier (Mir et al., 2020). The activity of catalase (CAT, E.C.1.11.1.6) was assessed by calculating the degradation of H_2_O_2_ in 3 min at 240 nm according to the procedure of Aebi (1984). Superoxide dismutase (SOD, E.C.1.15.1.1) activity was analyzed by calculating the drop-off in intensity of formazone, produced by the reaction of O2^–^ radicals and nitro-blue tetrazolium (NBT) dye as demonstrated earlier (Dhindsa et al., 1981). Peroxidase (POX, E.C.1.11.1.7) activity was measured by following the standard method developed by Sanchez et al. (1995), with slight modifications as described by Saleem and Fariduddin (2022).

### Cell Viability and Reactive Oxygen Species Localization in Roots

Cell viability in the roots was determined using a fluorescent dye (propidium iodide). Fluorescence was viewed using a confocal microscope (Zeiss, LSM 780, Tokyo, Japan). ROS accumulation in the cells was observed by sopping the roots in DCF-DA dye. The fluorescence was viewed using a confocal microscope (Zeiss, LSM 780, Tokyo, Japan).

### Stomatal Morphological Studies

Leaf stomata were studied using a method demonstrated by Talbot and White (2013). Fresh leaf samples were taken and immediately fixed with methanol before being immersed in ethanol. Dehydrated leaf samples were gold-coated, and stomatal morphology was observed using a scanning electron microscope (JEOL JSM–6510, Tokyo, Japan).

### Ultrastructural Analysis

The middle section of the leaf samples, devoid of the midrib, was selected and chopped into small disks. The samples were fixed with 4% glutaraldehyde buffer solution for 24 h at 4°C. Thereafter, the samples were carefully washed in a phosphate buffer (pH 7.4) and fixed again in 5% OsO_4_. The samples passed through the ethanol dilutions (50, 60, 70, 80, 90, and 100%, 15 min each), followed by acetone wash for 15 min, and were fixed in Epon 812 resin. Leaf sections were prepared with an LKB-V ultramicrotome. The samples were viewed under a transmission electron microscope (600-A-2, Hitachi, Japan).

### Element Status

Harvested plants were thoroughly washed with DDW followed by 0.5 M EDTA, and oven-dried for 48 h at 80°C. A dried sample (1 g) was crushed and digested in a solution containing HNO_3_ and HClO_4_ and heated on a hot plate until a transparent solution was obtained. The solution was cooled and diluted to 100 ml with DDW. The filtrate obtained was used to analyze elements with an atomic absorption spectrophotometer (Akram et al., 2015). Bioconcentration factor (BCF), bioaccumulation coefficient (BAC), and translocation factor (TF) were determined by the following equations:


$$\text{BCF}=\frac{\text{Cu}\,\mathrm{content}\,\mathrm{in}\,\mathrm{root}}{\mathrm{Cu}\,\mathrm{content}\,\mathrm{in}\,\mathrm{soil}}$$

$$\text{BAC}=\frac{\text{Cu}\,\mathrm{content}\,\mathrm{in}\,\mathrm{shoot}}{\mathrm{Cu}\,\mathrm{content}\,\mathrm{in}\,\mathrm{soil}}$$

$$\text{TF}=\frac{\text{Cu}\,\mathrm{content}\,\mathrm{in}\,\mathrm{shoot}}{\mathrm{Cu}\,\mathrm{content}\,\mathrm{in}\,\mathrm{root}}$$

Cu removal efficiency was calculated using the equation:


Removal⁢efficiency%

$$=\frac{\mathrm{Initial}\,\mathrm{content}\,\mathrm{of}\,\mathrm{Cu}\,\mathrm{in}\,\text{soil}-\mathrm{Final}\,\mathrm{content}\,\mathrm{of}\,\mathrm{Cu}\,\mathrm{in}\,\text{soil}}{\mathrm{Initial}\,\mathrm{content}\,\mathrm{of}\,\mathrm{Cu}\,\mathrm{in}\,\text{soil}}$$

### Statistical Analysis

SPSS ver. 20 for windows was used to analyze the significant difference at *p* ≤ 0.05 by analysis of variance (ANOVA) and Tukey tests. OriginPro was used for principal component analysis (PCA) and Pearson analysis.

## Results

### Tolerance Index of *Brassica juncea*

A decline in tolerance index (TiPH and TiVG) was observed in plants grown on Cu-amended soil. The highest decrease of 28.51% in TiPH and 19.55% in TiVG (Table 1) was observed in plants raised with 60 mg kg^–1^ of Cu in soil. On the other hand, the exogenous application of MEL exhibited positive effect and improved growth by improving plant tolerance index. The highest tolerance index over the control was reported in plants sprayed with 40 μM of MEL and was 25.3 and 57.07% in TiPH and TiVG, respectively. The application of MEL proved effective in improving and minimizing the damage induced by Cu stress (Table 1).

**TABLE 1:** Effect of different levels of soil applied Cu (0, 30, or 60 mg kg^–1^) and/or MEL (30, 40, or 50 μM) on tolerance index expressed in terms of plant height (TiPH), vegetative growth (TiVG), and RWC of *Brassica juncea* at 45 DAS.

Treatments	TiPH	TiVG	RWC%
Control	100 (72.69) ± 3.63^d^	100 (12.60) ± 0.60^e^	65.66 ± 1.24^de^
Cu (30 mg kg^–1^)	88.14 ± 3.20^e^	93.10 ± 0.56^f^	61.71 ± 1.17^f^
Cu (60 mg kg^–1^)	71.51 ± 2.60^f^	80.45 ± 0.49^g^	58.47 ± 1.11^g^
MEL (30 μM)	118.52 ± 4.38^b^	126.88 ± 0.74^c^	80.52 ± 1.52^bc^
MEL (40 μM)	125.30 ± 4.91^a^	157.05 ± 0.94^a^	87.73 ± 1.66^a^
MEL (50 μM)	115.97 ± 6.07^b^	142.05 ± 0.84^b^	83.55 ± 1.58^b^
Cu (30) + MEL (30 μM)	101.92 ± 3.37^d^	109.75 ± 0.62^de^	66.19 ± 1.25^d^
Cu (30) + MEL (40 μM)	112.65 ± 3.71^bc^	120.70 ± 0.65^d^	68.76 ± 1.30^d^
Cu (30) + MEL (50 μM)	105.44 ± 3.53^c^	113.33 ± 0.63^de^	66.98 ± 1.27^d^
Cu (60) + MEL (30 μM)	83.34 ± 2.76^ef^	91.37 ± 0.51^f^	63.80 ± 1.21^e^
Cu (60) + MEL (40 μM)	94.82 ± 3.16^de^	97.86 ± 0.54^ef^	66.04 ± 1.25^de^
Cu (60) + MEL (50 μM)	88.28 ± 2.97^e^	94.02 ± 0.53^f^	64.74 ± 1.22^e^

*Data shows the mean ± standard error. Same letters indicate that there is no significant difference at P < 0.05.*

### Status of Relative Water Content in Plants

Relative water content is the primary factor indicating plants’ water status and ability to survive under stress conditions. In the present study, all the Cu levels significantly reduced the RWC in plants compared to the control (Table 1). Plants raised under higher Cu level (60 mg kg^–1^) exhibited minimum RWC and was 10.95% less than that of the control. The maximum increase in RWC was observed in plants sprayed with MEL alone. Among the different levels sprayed, 40 μM of MEL efficiently increased RWC by 33.61% as compared to the control. Moreover, the follow-up treatment with MEL minimized the Cu-induced negative impact and improved the RWC content in Cu-stressed plants (Table 1).

### Gas Exchange Parameters and SPAD Value

All the levels of Cu significantly reduced the SPAD value, gas exchange parameters (*P_*N*_, g_*s*_, Ci*, and *E*), and leaf area as compared to control (Table 2). Plants raised with Cu (60 mg kg^–1^) exhibited a maximum reduction in all the aforementioned parameters that was 19.25% in the SPAD value, 25.69% in *P*_*N*_, 26.86% in *g*_*s*_, 29.03% in *C*_*i*_, 24.74% in *E*, and 18.81% in leaf area compared to the control. In contrast, MEL application improved the photosynthetic attributes. Among the different doses of MEL, 40 μM spray to the spray to the foliage proved more effective than the other concentrations, and increased the SPAD value by 21.54% in chlorophyll content, *P*_*N*_ by 40.1%, *g*_*s*_ by 52.23%, *C*_*i*_ by 29.35%, *E* by 24.74%, and leaf area by 23.27%. Moreover, the follow-up treatment with MEL of the Cu-stressed plants completely neutralized the toxic response imposed by Cu (30 mg kg^–1^), whereas partial restoration was observed in plants grown with Cu (60 mg kg^–1^)-amended soil (Table 2).

**TABLE 2:** Effect of different levels of soil applied Cu (0, 30, or 60 mg kg^–1^) and/or MEL (30, 40, or 50 μM) on SPAD chlorophyll values, stomatal conductance (*g*_*s*_) (mol m^–2^ s ^–1^), net photosynthetic rate (*P*_*N*_) (M CO_2_ m^–2^ s ^–1^), internal CO_2_ concentration (*C*_*i*_) (ppm), transpiration rate (*E*) (mol m^–2^ s ^–1^), and leaf area (cm^2^) of *Brassica juncea* at 45 DAS.

Treatments	SPAD	*gs*	*P*_*N*_	*C*_*i*_	*E*	Leaf area
Control	40.80 ± 0.77^cd^	0.067 ± 0.0025^c^	14.71 ± 0.28^c^	310 ± 5.86^d^	3.92 ± 0.074^d^	34.29 ± 0.65^cd^
Cu (30 mg kg^–1^)	36.87 ± 0.70^ef^	0.060 ± 0.0023^ef^	12.79 ± 0.24^d^	262 ± 4.96^g^	3.51 ± 0.066^fg^	31.89 ± 0.60^de^
Cu (60 mg kg^–1^)	33.04 ± 0.62^g^	0.049 ± 0.0019^h^	10.93 ± 0.21^f^	220 ± 4.16^i^	2.95 ± 0.056^h^	27.84 ± 0.53^f^
MEL (30 μM)	45.25 ± 0.86^b^	0.093 ± 0.0035^b^	18.55 ± 0.35^b^	352 ± 6.66^c^	4.32 ± 0.082^b^	38.56 ± 0.73^b^
MEL (40 μM)	49.59 ± 0.94^a^	0.102 ± 0.0039^a^	20.61 ± 0.39^a^	401 ± 7.59^a^	5.33 ± 0.101^a^	42.27 ± 0.80^a^
MEL (50 μM)	47.25 ± 0.89^ab^	0.097 ± 0.0037^ab^	19.44 ± 0.37^a^	371 ± 7.02^b^	4.79 ± 0.091^ab^	39.11 ± 0.74^b^
Cu (30) + MEL (30 μM)	39.60 ± 0.75^d^	0.065 ± 0.0025^de^	13.57 ± 0.26^cd^	288 ± 5.46^ef^	3.84 ± 0.073^fg^	35.08 ± 0.66^c^
Cu (30) + MEL (40 μM)	42.35 ± 0.80^c^	0.068 ± 0.0026^d^	14.85 ± 0.28^c^	312 ± 5.91^d^	4.05 ± 0.077^c^	35.95 ± 0.68^c^
Cu (30) + MEL (50 μM)	40.68 ± 0.77^cd^	0.066 ± 0.0025^d^	14.25 ± 0.27^cd^	296 ± 5.59^de^	3.91 ± 0.074^d^	35.48 ± 0.67^c^
Cu (60) + MEL (30 μM)	36.78 ± 0.70^ef^	0.055 ± 0.0021^f^	12.11 ± 0.23^de^	249 ± 4.70^h^	3.51 ± 0.066^fg^	32.62 ± 0.62^de^
Cu (60) + MEL (40 μM)	39.93 ± 0.76^d^	0.059 ± 0.0022^ef^	13.46 ± 0.25^d^	292 ± 5.51^e^	3.85 ± 0.073^de^	34.64 ± 0.66^cd^
Cu (60) + MEL (50 μM)	37.77 ± 0.71^e^	0.056 ± 0.0021^f^	12.58 ± 0.24^d^	268 ± 5.06^g^	3.61 ± 0.068^f^	33.65 ± 0.64^d^

*Data shows the mean ± standard error. Same letters indicate that there is no significant difference at P < 0.05.*

### Chlorophyll Fluorescence

All the concentrations of Cu decreased the chlorophyll fluorescence parameters *viz*, APSII, maximum PSII efficiency, ETR, and qP; however, NPQ was increased as compared to control (Table 3). The maximum decline was noted in plants raised with Cu 60 mg kg^–1^, and was 15.87% in APSII, 15.38% in maximum PSII efficiency, 18.05% in ETR, and 15.64% in PQ. However, NPQ was increased by 18.18%. In contrast, the exogenous application of MEL alone improved the parameters mentioned above. The utmost increase was noted by application of 40 μM of MEL, and the increase was 23.33% in APSII, 29.48% in maximum PSII efficiency, 9.25% in ETR, and 27.48% in PQ; contrastingly, NPQ was decreased by 16.66% as compared to the control. Moreover, the adverse effects imposed by Cu were neutralized by the follow-up treatment with MEL, and 40 μM of MEL as a foliar spray efficiently lowered the damage and restored the chlorophyll fluorescence under both stress and non-stress conditions (Table 3).

**TABLE 3:** Effect of different levels of soil applied Cu (0, 30, or 60 mg kg^–1^) and/or MEL (30, 40, or 50 μM) on maximum PSII efficiency (Max psII), actual PSII efficiency (Actual psII), electron transport rate (ET), photochemical quenching (qP), non-photochemical quenching (NPQ), and Rubisco activity (μmol CO_2_ protein min^–1^) of *Brassica juncea* at 45 DAS.

Treatments	Max psII	Actual psII	ET	qP	NPQ	Rubisco activity
Control	0.78 ± 0.015^bc^	0.63 ± 0.012^c^	216 ± 4.09^b^	0.735 ± 0.014^cd^	0.66 ± 0.012^cd^	49.10 ± 0.93^bc^
Cu (30 mg kg^–1^)	0.71 ± 0.013^d^	0.58 ± 0.011^d^	192 ± 3.63^d^	0.700 ± 0.013^d^	0.73 ± 0.014^b^	46.00 ± 0.87^d^
Cu (60 mg kg^–1^)	0.66 ± 0.012^de^	0.53 ± 0.010^e^	177 ± 3.35^e^	0.620 ± 0.012^e^	0.78 ± 0.015^a^	42.59 ± 0.81^f^
MEL (30 μM)	0.83 ± 0.017^b^	0.68 ± 0.013^b^	231 ± 4.37^a^	0.826 ± 0.016^b^	0.61 ± 0.012^e^	52.23 ± 0.99^ab^
MEL (40 μM)	0.97 ± 0.019^a^	0.77 ± 0.015^a^	236 ± 4.47^a^	0.937 ± 0.018^a^	0.55 ± 0.011^f^	54.50 ± 1.03^a^
MEL (50 μM)	0.92 ± 0.018^ab^	0.70 ± 0.013^b^	233 ± 4.41^a^	0.837 ± 0.016^b^	0.58 ± 0.012^ef^	52.29 ± 0.99^ab^
Cu (30) + MEL (30 μM)	0.76 ± 0.015^c^	0.63 ± 0.012^c^	206 ± 3.89^bc^	0.753 ± 0.014^cd^	0.65 ± 0.014^d^	49.14 ± 0.93^cd^
Cu (30) + MEL (40 μM)	0.83 ± 0.016^b^	0.68 ± 0.013^b^	229 ± 4.33^ab^	0.784 ± 0.015^c^	0.59 ± 0.013^ef^	51.31 ± 0.97^b^
Cu (30) + MEL (50 μM)	0.79 ± 0.015^bc^	0.66 ± 0.012^bc^	217 ± 4.11^b^	0.768 ± 0.015^c^	0.63 ± 0.013^de^	50.42 ± 0.95^bc^
Cu (60) + MEL (30 μM)	0.72 ± 0.014^d^	0.59 ± 0.011^d^	191 ± 3.62^d^	0.666 ± 0.013^de^	0.75 ± 0.015^ab^	45.19 ± 0.85^de^
Cu (60) + MEL (40 μM)	0.81 ± 0.016^bc^	0.67 ± 0.013^bc^	210 ± 3.97^bc^	0.720 ± 0.014^cd^	0.68 ± 0.014^c^	48.56 ± 0.92^c^
Cu (60) + MEL (50 μM)	0.76 ± 0.015^c^	0.62 ± 0.012^c^	203 ± 3.83^c^	0.694 ± 0.013^d^	0.72 ± 0.015^b^	46.58 ± 0.88^d^

*Data shows the mean ± standard error. Same letters indicate that there is no significant difference at P < 0.05.*

### Rubisco Activity

The activity of rubisco was decreased in plants raised with graded levels of Cu in a concentration-dependent manner (Table 3), and minimum activity was noted in plants raised with Cu 60 mg kg^–1^. On the other hand, exogenous application of MEL improved the activity of rubisco at all the tested concentrations. The maximum activity was observed in plants sprayed with MEL (40 μM), which was 10.99% more than the control. Moreover, the negative impact induced by Cu was minimized by the follow-up treatment of MEL, and the response was more significant in plants sprayed with 40 μM followed by 50 and 40 μM of MEL.

### Stomatal Morphology

**FIGURE 1:**
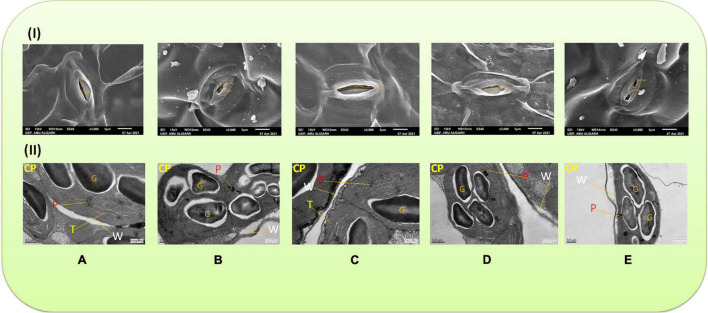
**(I)** Scanning electron microscope (SEM) images of stomata in 45 day-old leaves of *Brassica juncea* (L.) cv. Varuna raised with Cu and/or sprayed with melatonin (MEL) at 3,000× magnification. **(A)** Control, **(B)** Cu 60 mg kg^–1^, **(C)** MEL 40 μM, **(D)** Cu 30 + MEL (40 μM), and **(E)** Cu 60 + MEL (40 μM). **(II)** Transmission electron microscope (TEM) images of chloroplast in 45 day-old *B. juncea* (L.) cv. Varuna raised with Cu and/or sprayed with MEL. **(A)** Control, **(B)** Cu 60 mg kg^–1^, **(C)** MEL 40 μM, **(D)** Cu 30 + MEL (40 μM), and **(E)** Cu 60 + MEL (40 μM). CP, chloroplast; T, thylakoid; P, plastoglobuli; G, omniferous granules; W, cell wall.

Stomatal behavior was severely affected in the plants raised with Cu-amended soils. The minimum stomatal pore size was observed in the plants grown with 60 mg Cu kg^–1^ (Figure 1IB), whereas the widened stomatal aperture was observed in the plants sprayed with 40 μM of MEL (Figure 1IC). Moreover, exogenous application with MEL, particularly 40 μM of MEL, mitigated the toxic response of Cu and improved the size of stomatal aperture compared to the other concentrations (Figures 1IA–E).

### Ultrastructure Analysis

As evident in Figure 1IIA, the structure of chloroplast is intact with clear thylakoid lamella as well as the organization of grana. The chloroplast membrane and thylakoids were well-organized with less plastoglobuli. The difference between the control and MEL-treated plants chloroplast was minimal (Figure 1IIA,C). However, in the plants grown under excess Cu, the thylakoid membrane gets disintegrated, Chloroplast is not clearly visible (Figure 1IIB). The number of platoglobuli and osmophilic granules was higher in the chloroplast of the Cu-stressed plants. However, the damage induced by Cu was restored by the follow-up treatment of MEL, and noticeable restoration was observed in the Cu-stressed plants sprayed with 40 μM of MEL (Figures 1IID,E) in terms of chloroplast ultrastructure and organization, with lower number of plastoglobuli, and maintenance of the membrane structure.

### Total Soluble and Reducing Sugar and Starch Content

MEL application enhanced the sugar accumulation in plants compared to the non-melatonin-treated plants. Among the different levels of MEL, the spray of 40 μM proved more effective and improved the total soluble and reducing sugar and starch content by 26.41, 23.81, and 19.95%, respectively, as compared to the control (Table 4). In contrast, their content was reduced in the leaves of the plants grown with Cu-amended soil. The maximum reduction was observed in plants raised at higher levels of Cu (60 mg kg^–1^) and was 15.19, 14.32, and 38.51% less than the control. The ill-effect generated by both levels of Cu was mitigated by the foliar application of MEL, and the maximum positive effect was generated with 40 μM of MEL.

**TABLE 4:** Effect of different levels of soil applied Cu (0, 30, or 60 mg kg^–1^) and/or MEL (30, 40, or 50 μM) on total Soluble sugar (mg g^–1^ DW), reducing sugar (mg g^–1^ DW), glucose (mg g^–1^ FM), fructose (mg g^–1^ FM), sucrose (mg g^–1^ FM), and starch (mg g^–1^ FM) content in *Brassica juncea* at 45 DAS.

Treatments	Total sugar content	Reducing sugar	Glucose	Fructose	Sucrose	Starch
Control	139.00 ± 2.63^e^	12.50 ± 0.24^cd^	0.24 ± 0.0045^c^	0.171 ± 0.0032^e^	0.725 ± 0.0137^de^	15.89 ± 0.30^c^
Cu (30 mg kg^–1^)	124.90 ± 2.36^fg^	11.48 ± 0.22^de^	0.22 ± 0.0042^d^	0.160 ± 0.0030^f^	0.700 ± 0.0132^d^	13.05 ± 0.25^de^
Cu (60 mg kg^–1^)	117.88 ± 2.23^h^	10.71 ± 0.20^f^	0.16 ± 0.0030^g^	0.115 ± 0.0022^j^	0.608 ± 0.0115^f^	9.77 ± 0.18^g^
MEL (30 μM)	160.10 ± 3.03^bc^	14.46 ± 0.27^b^	0.25 ± 0.0048^b^	0.207 ± 0.0039^bc^	0.779 ± 0.0147^bc^	16.96 ± 0.32^b^
MEL (40 μM)	175.71 ± 3.32^a^	15.48 ± 0.29^a^	0.31 ± 0.0058^a^	0.252 ± 0.0048^a^	0.834 ± 0.0158^a^	19.06 ± 0.36^a^
MEL (50 μM)	166.54 ± 3.15^ab^	14.73 ± 0.28^b^	0.27 ± 0.0052^b^	0.219 ± 0.0042^b^	0.797 ± 0.0151^b^	17.87 ± 0.34^b^
Cu (30) + MEL (30 μM)	139.20 ± 2.63^e^	12.22 ± 0.23^cd^	0.24 ± 0.0045^c^	0.176 ± 0.0033^de^	0.747 ± 0.0141^d^	15.83 ± 0.30^c^
Cu (30) + MEL (40 μM)	150.73 ± 2.85^d^	13.14 ± 0.25^c^	0.25 ± 0.0048^b^	0.186 ± 0.0035^d^	0.764 ± 0.0144^c^	16.97 ± 0.32^b^
Cu (30) + MEL (50 μM)	143.43 ± 2.71^de^	12.55 ± 0.24^cd^	0.25 ± 0.0047^b^	0.179 ± 0.0034^de^	0.754 ± 0.0143^cd^	16.58 ± 0.31^c^
Cu (60) + MEL (30 μM)	129.33 ± 2.45^f^	11.36 ± 0.21^e^	0.18 ± 0.0033^f^	0.130 ± 0.0025^i^	0.658 ± 0.0125^ef^	11.71 ± 0.22^f^
Cu (60) + MEL (40 μM)	135.62 ± 2.57^ef^	12.11 ± 0.23^d^	0.21 ± 0.0039^de^	0.150 ± 0.0028^g^	0.691 ± 0.0131^de^	15.07 ± 0.29^cd^
Cu (60) + MEL (50 μM)	130.37 ± 2.47^f^	11.51 ± 0.22^de^	0.19 ± 0.0037^f^	0.141 ± 0.0027^gh^	0.674 ± 0.0127^e^	13.60 ± 0.26^d^

*Data shows the mean ± standard error. Same letters indicate that there is no significant difference at P < 0.05.*

### Glucose, Sucrose, and Fructose Content

A maximum level of glucose, sucrose, and fructose was noted in the leaves of the plants sprayed with 40 μM of MEL, being 29.63, 15.02, and 47.28% more than that of the control (Table 4). However, the content was decreased in the leaves of the plants raised with Cu in a concentration-dependent manner. Moreover, the adverse effect was overcome by the foliar application of MEL, with the maximum being 40 μM.

### Hexokinase, Fumarase, and Succinate Dehydrogenase Activities

The activity of carbohydrate metabolism enzymes increases in the leaves of the plants grown on Cu-amended soil and were further enhanced by the foliar application of MEL. The maximum enhancement in the activity of HXK, FH, and SDH was observed in the plants raised in Cu (60 mg kg^–1^) and sprayed with 40 mM of MEL, which was 51.29% in HXK, 46.71% in FH and 48.46% in SDH, more than that of the control (Table 5).

**TABLE 5:** Effect of different levels of soil applied Cu (0, 30, or 60 mg kg^–1^) and MEL (30, 40, or 50 μM) on, leaf NR [n mole NO_2_ g^–1^ (FM) s^–1^], CA [mol (CO_2_) kg^–1^ (leaf FM) s^–1^], HXK (U mg^–1^ protein), FUM (μ mol mL^–1^ fumarate), SDH (μg dye reduced g^–1^ sample) activities and electrolyte leakage (%) in *Brassica juncea* at 45 DAS.

Treatments	NR	CA	HXK	FUM	SDH	EL
Control	423.10 ± 8.00^d^	2.32 ± 0.044^de^	0.067 ± 0.0025^f^	19.21 ± 0.73^f^	0.126 ± 0.0048^g^	5.28 ± 0.20^ef^
Cu (30 mg kg^–1^)	387.10 ± 7.32^e^	2.08 ± 0.039^f^	0.073 ± 0.0028^e^	21.86 ± 0.83^e^	0.15 ± 0.0057^e^	5.86 ± 0.22^d^
Cu (60 mg kg^–1^)	349.52 ± 6.61^f^	1.89 ± 0.036^g^	0.086 ± 0.0033^cd^	25.37 ± 0.96^bc^	0.168 ± 0.0064^cd^	8.99 ± 0.34^a^
MEL (30 μM)	467.45 ± 8.84^bc^	2.62 ± 0.050^c^	0.075 ± 0.0028^e^	22.39 ± 0.85^d^	0.146 ± 0.0055^f^	4.96 ± 0.19^f^
MEL (40 μM)	530.15 ± 10.03^a^	3.00 ± 0.057^a^	0.087 ± 0.0033^cd^	26.07 ± 0.99^bc^	0.171 ± 0.0065^bc^	3.70 ± 0.14^g^
MEL (50 μM)	495.72 ± 9.38^b^	2.87 ± 0.054^ab^	0.082 ± 0.0031^d^	23.66 ± 0.89^d^	0.163 ± 0.0062^d^	4.19 ± 0.16^g^
Cu (30) + MEL (30 μM)	431.19 ± 8.16^d^	2.30 ± 0.043^de^	0.083 ± 0.0031^d^	23.12 ± 0.87^d^	0.167 ± 0.0063^cd^	5.89 ± 0.22^d^
Cu (30) + MEL (40 μM)	456.82 ± 8.64^c^	2.52 ± 0.048^cd^	0.093 ± 0.0035^bc^	26.39 ± 1.00^b^	0.186 ± 0.0070^a^	5.54 ± 0.21^e^
Cu (30) + MEL (50 μM)	442.19 ± 8.36^cd^	2.42 ± 0.046^d^	0.088 ± 0.0033^cd^	25.18 ± 0.95^bc^	0.173 ± 0.0066^bc^	5.66 ± 0.21^e^
Cu (60) + MEL (30 μM)	415.08 ± 7.85^de^	2.10 ± 0.040^ef^	0.091 ± 0.0034^c^	25.31 ± 0.96^bc^	0.170 ± 0.0064^bc^	8.34 ± 0.32^a^
Cu (60) + MEL (40 μM)	439.12 ± 8.31^cd^	2.28 ± 0.043^de^	0.099 ± 0.0038^a^	28.18 ± 1.07^a^	0.187 ± 0.0071^a^	6.28 ± 0.24^c^
Cu (60) + MEL (50 μM)	431.37 ± 8.16^d^	2.18 ± 0.041^e^	0.095 ± 0.0036^b^	26.32 ± 1.00^b^	0.178 ± 0.0067^b^	7.20 ± 0.27^b^

*Data shows the mean ± standard error. Same letters indicate that there is no significant difference at P < 0.05.*

### Nitrate Reductase and Carbonic Anhydrase

The activity of nitrate reductase (NR) and carbonic anhydrase (CA) was significantly reduced in the Cu-stressed plants. The minimum activity of NR and CA was observed in the leaves of the plants raised with Cu (60 mg kg^–1^) and was 17.39 and 18.53% less than in the control, whereas the application of MEL as a foliar spray improved the activity of both enzymes, and maximum enhancement was generated with 40 μM of MEL (Table 5). Additionally, the ill-effect generated by Cu was reduced by the foliar application of MEL, particularly by the spray of 40 μM of MEL.

### Enzymatic and Non-enzymatic Antioxidants

An increase in the level of enzymatic (SOD, CAT, and POX) and non-enzymatic (GSH and proline) antioxidants was observed in the plants grown on Cu-amended soil. Their activities were further improved by the foliar spray of MEL (Table 6). The maximum enhancement in the activity of antioxidants was observed in the plants grown with Cu (60 mg kg^–1^) and sprayed with 40 μM of MEL, which was 33.08% in SOD, 31.13% in CAT, 44.98% in POX, 36.82% in GSH and 46.05% in proline content, more than that of the control. Forty μM of MEL proved most efficient in improving antioxidant levels under stress and normal conditions (Table 6).

**TABLE 6:** Effect of different levels of soil applied Cu (0, 30, or 60 mg kg^–1^) and/or MEL (30, 40, or 50 μM) on leaf peroxidase (units g^–1^ FM), catalase (mM H_2_O_2_ decomposed g^–1^ FM), superoxide dismutase (unit g^–1^ FM), glutathione (nmol g^–1^ FW), and proline (mg g^–1^ FM) content of *Brassica juncea* at 45 DAS.

Treatments	Peroxidase	Catalase	Superoxide-dismutase	Glutathione	Proline
Control	12.16 ± 0.23^g^	424.13 ± 8.02^f^	136 ± 2.57^f^	380 ± 7.19^g^	15.57 ± 0.29^f^
Cu (30 mg kg^–1^)	13.65 ± 0.26^ef^	452.50 ± 8.56^e^	152 ± 2.87^de^	402 ± 7.60^f^	17.36 ± 0.33^e^
Cu (60 mg kg^–1^)	14.68 ± 0.28^d^	488.56 ± 9.24^de^	163. ± 3.08^c^	443 ± 8.38^e^	18.95 ± 0.36^cd^
MEL (30 μM)	14.21 ± 0.27^g^	482.92 ± 9.13^de^	158 ± 3.00^d^	413 ± 7.81^f^	17.12 ± 0.32^e^
MEL (40 μM)	15.28 ± 0.29^c^	554.52 ± 10.68^a^	167 ± 3.16^bc^	461 ± 8.72^cd^	19.13 ± 0.36^cd^
MEL (50 μM)	14.73 ± 0.28^d^	527.28 ± 9.97^bc^	160 ± 3.04^cd^	439 ± 8.30^e^	18.02 ± 0.34^de^
Cu (30) + MEL (30 μM)	14.52 ± 0.27^de^	506.00 ± 9.57^d^	163 ± 3.08^c^	434 ± 8.20^e^	18.65 ± 0.35^d^
Cu (30) + MEL (40 μM)	16.38 ± 0.31^b^	533.19 ± 10.08^b^	170 ± 3.22^b^	497 ± 9.41^b^	20.44 ± 0.39^b^
Cu (30) + MEL (50 μM)	15.30 ± 0.29^c^	522.05 ± 9.87^c^	166 ± 3.14^bc^	474 ± 8.97^c^	19.87 ± 0.38^bc^
Cu (60) + MEL (30 μM)	16.67 ± 0.32^ab^	535.59 ± 10.13^b^	166 ± 3.14^bc^	472 ± 8.93^c^	20.19 ± 0.38^b^
Cu (60) + MEL (40 μM)	17.63 ± 0.33^a^	556.33 ± 10.52^a^	181 ± 3.44^a^	520 ± 9.84^a^	22.74 ± 0.43^a^
Cu (60) + MEL (50 μM)	16.73 ± 0.32^ab^	542.14 ± 10.25^b^	173 ± 3.28^b^	495 ± 9.37^b^	21.00 ± 0.40^b^

*Data shows the mean ± standard error. Same letters indicate that there is no significant difference at P < 0.05.*

### Oxidative Stress Biomarkers

**FIGURE 2:**
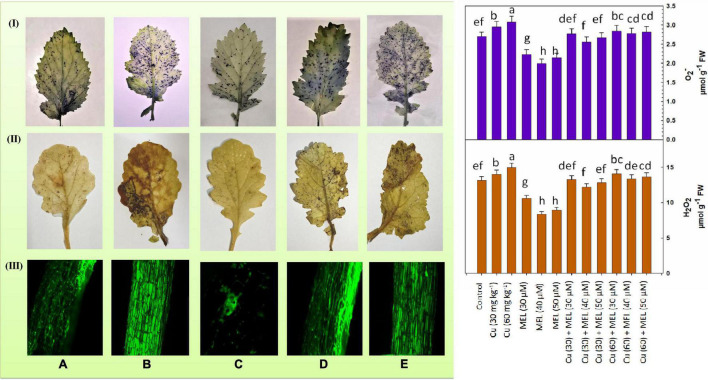
**(I)** Superoxide anion content and histochemical localization of O_2_^–^ in the leaves of 45-day-old *B. juncea* (L.) cv. Varuna raised with Cu and/or sprayed with MEL. **(A)** Control, **(B)** Cu 60 mg kg^–1^, **(C)** MEL 40 μM, **(D)** Cu 30 + MEL (40 μM), and **(E)** Cu 60 + MEL (40 μM). Data show the mean ± standard error, and same letters indicate no significant difference at *P* < 0.05. **(II)** Hydrogen peroxide content and histochemical localization of H_2_O_2_ in the leaves of 45-day-old *B. juncea* (L.) cv. Varuna raised with Cu and/or sprayed with MEL. **(A)** Control, **(B)** Cu 60 mg kg^–1^, **(C)** MEL μM, **(D)** Cu 30 + MEL (40 μM), and **(E)** Cu 60 + MEL (40 μM). Data show the mean ± standard error, and same letters indicate no significant difference at *P* < 0.05. **(III)** Reactive oxygen species (ROS) localization in the roots of 45-day-old *B. juncea* (L.) cv. Varuna raised with Cu and/or sprayed with MEL. **(A)** Control, **(B)** Cu 60 mg kg^–1^, **(C)** MEL μM, **(D)** Cu 30 + MEL (40 μM), and **(E)** Cu 60 + MEL (40 μM).

Figure 2 clearly indicates that Cu triggered the accumulation of O_2_^–^ and H_2_O_2_ in plants in a dose-dependent manner. The highest accumulation of O_2_^–^ and H_2_O_2_ was observed in plants grown with Cu (60 mg kg^–1^) on soil and was 14.07 and 13.6% more than that of the control. On the other hand, foliar application of MEL decreased the production of O_2_^–^ and H_2_O_2_, and minimum O_2_^–^ and H_2_O_2_ content was observed in the plants sprayed with 40 μM of MEL, which was 26.08 and 10.89% less than the control. Moreover, MEL, particularly 40 μM of MEL, proved effective in mitigating the toxic ROS levels in the Cu-stressed plants and decreased the level of O_2_^–^ and H_2_O_2_ to a remarkable level as compared to the non-treated plants. The observations were further validated by the histochemical localization of O_2_^–^ (Figure 2I) and H_2_O_2_ (Figure 2II), where the unstressed plants showed less staining; however, intensive staining was observed in the plants raised with higher levels of Cu. The observations demonstrated that both O_2_^–^ and H_2_O_2_ levels declined further by the foliar application of MEL, as clearly seen in Figures 2I,II.

Detection of ROS in the roots was conducted using the DCF-DA fluorescent dye and viewed under a confocal microscope. DCF-DA reacts with ROS molecules and emits green fluorescence in cells. Maximum green fluorescence indicates more ROS accumulation in cells. As evident in Figure 2III, the maximum green fluorescence is observed in root samples of the plants grown with Cu (60 mg kg^–1^), while minimum green fluorescence is seen in the plants treated with 40 μM of MEL. Interestingly, MEL application to the Cu-stressed plants reduced ROS accumulation in the roots, as evidenced by decreased fluorescence in the root samples of plants sprayed with MEL compared to the non-MEL-treated plants (Figures 2IIIC,D).

### Membrane Stability

**FIGURE 3:**
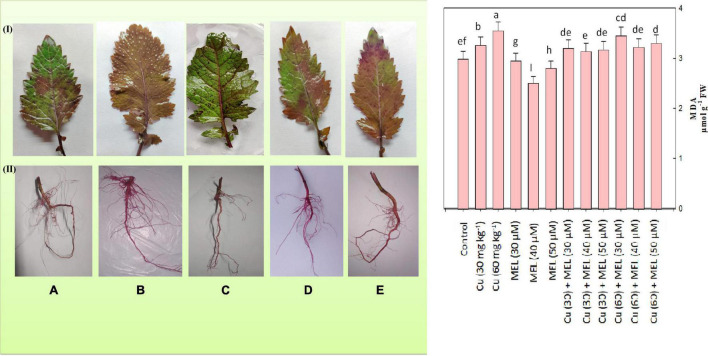
Malondialdehyde content and histochemical localization of lipid peroxidation in the **(I)** leaves and **(II)** roots of 45-day-old *B. juncea* (L.) cv. Varuna raised with Cu and/or sprayed with MEL. **(A)** Control, **(B)** Cu 60 mg kg^–1^, **(C)** MEL μM, **(D)** Cu 30 + MEL (40 μM), and **(E)** Cu 60 + MEL (40 μM). Data show the mean ± standard error, and same letters indicate no significant difference at *P* < 0.05.

To evaluate the effect of Cu and MEL alone as well as in combination on membrane integrity, MDA and EL were determined. The result indicated that both levels of Cu elevated the MDA (Figure 3) and EL content (Table 5), and maximum values was noted in plants raised with 60 mg of Cu kg^–1^, which was 18.73 and 70.26% more than that of the control. On the other hand, the foliar application of MEL decreased the MDA and EL content, and lowest values were observed in the plants sprayed with 40 μM of MEL. These observations were further validated by the histochemical localization of lipid peroxidation in roots and leaves, the higher the pink color intensity, the more the lipid peroxidation. The lower intensity of the pink color was seen in the plants treated with MEL (Figure 3C), whereas the maximum pink color was observed in the plant samples grown in Cu (60 mg kg^–1^, Figure 3B).

### Cell Death

**FIGURE 4:**
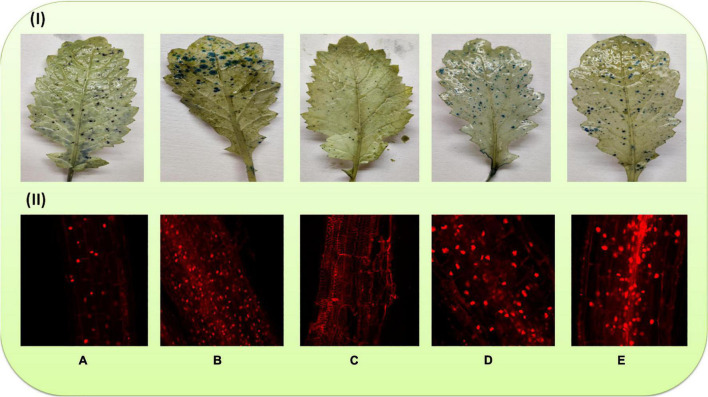
Cell death in the leaves and roots of 45-day-old *B. juncea* (L.) cv. Varuna raised with Cu and/or sprayed with MEL. **(A)** Control, **(B)** Cu 60 mg kg^–1^, **(C)** MEL μM, **(D)** Cu 30 + MEL (40 μM), and **(E)** Cu 60 + MEL (40 μM).

Death of cells in the leaves was observed histochemically by trypan blue staining. As evident in Figure 4I, the density of blue spots was more in the leaves of plants raised with Cu (60 mg kg^–1^)-amended soil (Figure 4IB), demonstrating more numbers of dead cells, whereas a minimum number of blue spots was observed in the plants sprayed with 40 μM of MEL alone (Figure 4IC). Moreover, the number of spots was noticeably reduced in the plants raised on Cu-amended soil and sprayed with MEL (Figures 4ID,E).

Cell death in the roots was observed using propodium iodide. The more the numbers of red fluorescent nuclei, the more the dead cells. Figures 4A,C demonstrates that the foliar application of MEL decreased the number of red fluorescent nuclei compared to the non-treated plants. In contrast, maximum red nuclei were visible in the plants grown on soil amended with 60 mg kg^–1^ of Cu (Figure 4IIB). However, the number of fluorescent nuclei was noticeably decreased in the Cu-stressed plants sprayed with MEL, particularly with 40 μM of MEL (Figures 4IID,E).

### Nutrient Composition

The results demonstrated that higher level of Cu considerably decreased the N, P and K content in plants compared to the control (Table 7). The minimum N, P, and K content was observed in plants grown with Cu (60 mg kg^–1^) and was 16.54 (N), 14.46 (P), and 15.35% (K) less than the control. On the other hand, MEL application alone improved the nutrient levels in the plants. Among the various sprayed levels, 40 μM of MEL generated maximum enrichment in nutrient levels and was 11.73 (N), 16.67 (P), and 22.83% (K) more than the control plants. Moreover, follow-up treatment with MEL mitigated Cu-induced nutrient imbalance, and maximum restoration in nutrient status was observed in the plants sprayed with 40 μM of MEL (Table 7).

**TABLE 7:** Effect of different levels of soil applied Cu (0, 30 or 60 mg kg^–1^) and/or MEL (30, 40 or 50 μM) on nitrogen content (% DW), potassium content (% DW), and phosphorus content (% DW) of *Brassica juncea* at 45 DAS.

Treatments	N content	P content	K content
Control	2.60 ± 0.049^c^	2.49 ± 0.047^de^	0.23 ± 0.0043^c^
Cu (30 mg kg^–1^)	2.40 ± 0.045^d^	2.36 ± 0.045^ef^	0.22 ± 0.0041^d^
Cu (60 mg kg^–1^)	2.17 ± 0.041^f^	2.13 ± 0.040^g^	0.19 ± 0.0037^g^
MEL (30 μM)	2.76 ± 0.052^ab^	2.68 ± 0.051^bc^	0.25 ± 0.0046^b^
MEL (40 μM)	2.91 ± 0.055^a^	2.91 ± 0.055^a^	0.28 ± 0.0053^a^
MEL (50 μM)	2.80 ± 0.053^ab^	2.78 ± 0.053^b^	0.25 ± 0.0048^b^
Cu (30) + MEL (30 μM)	2.60 ± 0.049^bc^	2.51 ± 0.047^d^	0.23 ± 0.0043^c^
Cu (30) + MEL (40 μM)	2.71 ± 0.051^b^	2.63 ± 0.050^c^	0.24 ± 0.0046^bc^
Cu (30) + MEL (50 μM)	2.64 ± 0.050^c^	2.56 ± 0.048^cd^	0.23 ± 0.0044^c^
Cu (60) + MEL (30 μM)	2.36 ± 0.045^de^	2.33 ± 0.044^f^	0.21 ± 0.0039^de^
Cu (60) + MEL (40 μM)	2.54 ± 0.048^c^	2.46 ± 0.046^de^	0.22 ± 0.0042^d^
Cu (60) + MEL (50 μM)	2.46 ± 0.046^d^	2.38 ± 0.045^ef^	0.21 ± 0.0040^de^

*Data shows the mean ± standard error. Same letters indicate that there is no significant difference at P < 0.05.*

### Copper Accumulation and Remediation

The accumulation of Cu in the plants increased as its concentrations increased in the soil and was further increased by the foliar application of MEL. The highest Cu accumulation was observed in plants raised with higher Cu concentrations and sprayed with 40 μM of MEL, and had 4.01 and 4.06 μg g^–1^ Cu on a dry mass basis in the roots and shoots, respectively, compared with 0.57 and 0.4 μg g^–1^ in roots and shoots of the control (Table 8). A slight increase in Cu accumulation over the control was observed in the plants sprayed with MEL alone. Moreover, MEL supplementation enhanced the translocation factor (>1) in all the plants raised under Cu (30 mg kg^–1^) and was near 1 (≤1) in the plants raised under Cu 60 mg kg^–1^. In addition, BCF and BAC were also improved in the plants treated with MEL. The result also suggested that MEL significantly enhanced Cu removal efficiency in the test plants (Table 8).

**TABLE 8:** Effect of different levels of soil applied Cu (0, 30, or 60 mg kg^–1^) and/or MEL (30, 60, and 90 μM) on copper content (root and shoot) (μg g^–1^ DM), translocation factor (%), BCF (bio-concentration factor), BAC (bioaccumulation coefficient), and removal efficiency (%) of *Brassica juncea* at 45 DAS.

Treatments	Root Cu content	Shoot Cu content	Translocation factor	BCF	BAC	Removal efficiency (%)
Control	0.57 ± 0.010^i^	0.40 ± 0.007^j^	0.73 ± 0.064^e^	0.20 ± 0.003^f^	0.32 ± 0.003^e^	17.19 ± 0.86^f^
Cu (30 mg kg^–1^)	2.35 ± 0.044^g^	0.83 ± 0.02^h^	0.36 ± 0.035^f^	0.28 ± 0.004^d^	0.23 ±0.002^f^	16.64 ± 0.83^f^
Cu 60 mg kg^–1^)	3.73 ± 0.070^c^	1.35 ± 0.03^g^	0.37 ± 0.035^f^	0.24 ± 0.003 ^de^	0.20 ± 0.002^f^	14.36 ± 0.72^g^
IAA (10^–10^ M)	0.59 ± 0.011^hi^	0.64 ± 0.009^i^	1.08 ± 0.062^bc^	0.27 ±0.004 ^d^	0.61 ± 0.003^d^	29.09 ± 1.40^de^
IAA (10^–8^ M)	0.66 ± 0.012^h^	0.82 ± 0.010^h^	1.23 ± 0.070^a^	0.39 ± 0.005^a^	0.97 ± 0.005^a^	45.06 ± 2.16^a^
IAA (10^–6^ M)	0.62 ± 0.011^h^	0.71 ± 0.011^i^	1.15 ± 0.068 ^b^	0.32 ± 0.004^c^	0.77 ± 0.004^b^	36.16 ± 1.74 ^c^
Cu (30)+ IAA (10^–10^ M)	2.41± 0.042^f^	2.64 ± 0.016^f^	1.09 ± 0.033^cd^	0.32 ± 0.004^c^	0.75 ± 0.002^c^	35.58 ± 1.71^d^
Cu (30)+ IAA (10^–8^ M)	2.67 ± 0.036^de^	3.10 ±0.016^d^	1.16 ± 0.032^b^	0.38 ± 0.005^a^	0.90 ± 0.002^ab^	42.46 ± 2.04^b^
Cu (30)+ IAA (10^–6^ M)	2.58 ± 0.039^e^	2.85 ± 0.017^e^	1.10 ± 0.031^b^	0.36 ± 0.005^b^	0.82 ± 0.002^b^	39.06 ± 1.88^c^
Cu (60)+ IAA (10^–10^ M)	3.87 ± 0.069^c^	3.58 ± 0.026^b^	0.92 ± 0.034^d^	0.26 ± 0.003^de^	0.56 ± 0.003^f^	27.15 ± 1.30 ^e^
Cu (60)+ IAA (10^–8^ M)	4.01 ± 0.057^a^	4.06 ± 0.020^a^	1.01 ± 0.031^c^	0.31 ± 0.004^c^	0.66 ± 0.004^c^	31.73 ± 1.52^d^
Cu (60)+ IAA (10^–6^ M)	3.92 ± 0.065^b^	3.80 ± 0.025^b^	0.97 ± 0.032^c^	0.29 ± 0.004^d^	0.60 ± 0.003^d^	29.37 ± 1.41^de^

*Data shows the mean ± standard error. Same letters indicate that there is no significant difference at P < 0.05.*

## Discussion

In this study, we have provided an insight into how melatonin regulates growth and development in *Brassica juncea* exposed to excessive Cu levels. Prolonged Cu stress resulted in decrease in tolerance index (TiPH and TiVG) in a dose-dependent manner (Table 1). This is possibly an expression of increase in stress biomarkers (ROS, MDA, and EL) (Figures 2, 3 and Table 6), which directly interfere with physio-biochemical attributes and inhibited photosynthetic functions (Tables 2, 3), nutrient assimilation (Table 7), and sugar metabolism (Table 4). On the other hand, the exogenous spray of MEL improved the tolerance index in the plants, demonstrating the ameliorative action of MEL in mitigating Cu-imposed toxicity. Moreover, MEL improved the antioxidant capacity and consequent decreases the level of ROS and MDA, thus protecting the membrane integrity and organization of chloroplast and stomata, thereby enhancing the photosynthetic rate and sugar accumulation and, subsequently, the growth of and biomass production in the Cu-stressed plants. In addition, MEL maintains the robust root system, enhances root growth (Qiao et al., 2019), and maintains the membrane integrity of cells during stress conditions (Yang et al., 2022). In accordance with this, the results also suggested the positive role of MEL in maintaining the root architecture and cell viability (Figure 4) in metal-stressed plants. More importantly, the MEL-induced refurbishment of RWC in the Cu-stressed plants suggested its membrane-protecting and osmoprotective role in plants (Table 1). The present study signifies that exogenous application of MEL could be beneficial for plants coping with heavy metal contamination.

Gas exchange attributes photosynthetic fluorescence parameters, rubisco activity, and SPAD values are noted to decrease in Cu-treated plants in a dose-dependent manner. Moreover, Cu reduced the stomatal size and caused severe damage to the ultrastructure of chloroplast (Figure 1). These observations are in conformity with others (Mir et al., 2021b; Rather et al., 2022). Excessive accumulation of Cu in plants deforms the integrity and fluidity of the thylakoid membrane, downregulates the genes involved in chlorophyll biosynthesis, and reduces photosynthetic efficiency (Parveen et al., 2020). Moreover, the decrease in *P*_*N*_ and *E* with reduced quantum yield of photochemistry under excessive Cu levels might be due to the inhibition of enzymatic processes involved in the Calvin cycle as well as reduction in carbon metabolism and leaf water use efficiency (Ouzounidou and Constantinidou, 1999). In addition, the restricted mineral uptake under higher Cu levels affects leaf development and interferes with stomatal movements and energy metabolism (Mir et al., 2021). However, the follow-up treatment with MEL mitigated the Cu-induced toxicity by improving the photosynthetic rate and gas exchange parameters and pigment composition apart from shielding the ultrastructure and morphology of photosynthetic machinery in stressed plants. This improvement is ascribed to the MEL’s potential of maintaining ionic homeostasis, upregulating the activity of CA and other enzymes involved in photosynthesis, and decreasing reactive oxygen radicals and lipid peroxidases (Mir et al., 2021). Moreover, MEL downregulates the activity of chlorophyll catabolic enzymes such as pheophorbide, an oxygenase (PAO), pheophytinase (PPH), and chlorophyllase (CLH), and inhibits the expression of *BoNOL*, *BoNYC1*, *BoRCCR, BoPPH*, *BoCLH*, *BoPAO*, and *BoSGR1*, which are involved in chlorophyll catabolism (Wu et al., 2021).

Copper catalyzes the production of different ROS molecules, thereby initiating oxidative stress in plant cells (Kumar et al., 2021; Rather et al., 2022; Mir et al., 2022). ROS, in turn, trigger lipid peroxidation and oxidation of nucleic acids and proteins, causing their structural alterations and cellular dysfunction in plants (Huang et al., 2019). In the present study, the MDA and EL content increased considerably (Figure 3 and Table 5), triggered by the enhanced production of O_2_^–^ and H_2_O_2_ (Figure 2II) in the Cu-stressed plants. However, these oxidative responses were reduced by the foliar application of MEL (Figures 2I–III), Table 5), indicating the protective role of MEL in plants exposed to Cu stress. Several studies also reported that MEL decreased the ROS and lipid peroxidase levels in plants exposed to multiple stresses (Wang et al., 2013; Zhang et al., 2017; Ulhassan et al., 2019). In response to these reactive oxygen radicals and lipid peroxidases, plants have evolved an intricate antioxidant system to deal with these harmful oxygen radicals and mitigate the toxic influence of Cu. This is naturally regulated by enhancing the activity of antioxidative enzymes such as SOD, CAT, and POX. For example, SOD is the primary defense against O_2_^–^ radicals. It catalyzes the conversion of O_2_^–^ to H_2_O_2_, which is consequently condensed to H_2_O by POX (Hasanuzzaman et al., 2020). In our study, all the treatments upregulated the enzymatic (CAT, SOD, and POX) and non-enzymatic (GSH and proline) antioxidants. These oxidative scavengers were further upregulated by the exogenous application of MEL (Table 6). Maximum antioxidant levels were reported in the Cu (60 mg kg^–1^)-stressed plants supplemented with 40 μM of MEL. Such a defense capacity was more apparent and may be directly correlated to higher ROS, MDA, and EL content, and MEL’s ability to modulate the expression of key genes involved in synthesis of antioxidants (Sunyer-Figueres et al., 2020). MEL also regulates the ascorbate–glutathione cycle (APX, MDAR, and GSH), thus ameliorating the free radical damage caused by toxic levels of Cu in plants (Khan et al., 2020).

Excessive Cu directly affects nitrogen metabolism by inhibiting the activity of nitrate reductase, which is essential for nitrogen assimilation in plants (Hippler et al., 2018). In this study, Cu inhibited the activity of NR and N assimilation in the stressed plants, and maximum reduction was observed in the plants raised with Cu (60 mg kg^–1^). The deduction may be due to reduced uptake of N by the roots, dysfunction of the enzyme under excess Cu conditions, alterations in membrane fluidity and composition (Llorens et al., 2000) and root architecture (Yusefi-Tanha et al., 2020), and metabolic dysfunction of the protein enzyme involved in nitrogen assimilation (Hippler et al., 2018). However, the application of MEL mitigated the adverse effect induced by Cu and improved the activity of NR and N content (Table 7). Additionally, excessive Cu accumulation in the plants decreased the activity of CA (Table 5), which is seemingly because of its intervention with the organization and permeability of the plasma membrane, thereby causing nutrient imbalance, especially the limited uptake of Zn ion necessary for CA regulation (Hayat et al., 2007; Islam et al., 2021). However, the inhibitory effect of Cu on the activity of CA was decreased with the exogenous application of MEL. Our study demonstrated that the application of 40 μM of MEL efficiently mitigated the damage caused by excessive Cu levels and upregulated the activity of CA in the Cu-stressed plants, possibly by improvement in nutrients status (Table 7) and internal CO_2_ levels, and reduced accumulation of ROS, MDA, and EL content (Figures 2, 3).

Heavy metals interfere with sugar accumulation and distribution in plants. Many studies suggested a reduction in total sugar content in plants exposed to heavy metals (Verma and Dubey, 2001; Devi et al., 2013). The present observations also revealed that total sugar, reducing sugar, and starch content as well as glucose, sucrose, and fructose content was decreased in the Cu-stressed plants (Table 4), which was seemingly due to limited mineral absorption, especially the restricted uptake of Fe, as well as altered membrane structure and permeability (Patsikka, 2002). Moreover, decrease in photosynthetic rate and pigment composition and higher accumulation of MDA and ROS may perhaps be two of the causes of reduced sugar metabolism in Cu-stressed plants (Mir et al., 2021a). In accordance with our results, Dowidar et al. (2013) reported a decrease in sucrose and starch content in *Trigonella foenum-graecum* exposed to Cu stress. In contrast, the exogenous application of MEL improved the sugar levels (Table 4), indicating the protective role of MEL in mitigating the toxic effects imposed by Cu. Several studies suggested that MEL increased the expression of genes involved in photosynthesis, carbohydrate metabolism, Krebs cycle, and other metabolic pathways in plants (Samanta et al., 2020; Chen et al., 2021). Moreover, MEL application maintained nutrient homeostasis by restoring the root architecture and cell viability in Cu-stressed plants (Altaf et al., 2020). Mir et al. (2020) also revealed that foliar spray of MEL could be an alternative approach to maintain nutrient homeostasis for proper growth and development in plants, corroborating the previous findings.

Copper accumulation in roots and shoots increased in a dose-dependent manner (Table 8). Under normal and stress conditions, accumulation was more in roots, a little was transported to above-ground tissues and organs, and TF was found to be <1 in all the plants. On the other hand, the application of MEL, particularly 40 μM, significantly improved the Cu translocation from root to shoot. The MEL treated plants raised with Cu (30 mg kg^–1^) exhibited a TF > 1 at whereas its value was <1 in plants grown with Cu 60 mg kg^–1^ of soil. Hence, the technique may be applied to shift the metals from roots to shoots in reclamation of Cu-contaminated soils. Similar observations have also been observed in *B. juncea* exposed to Cu (Napoli et al., 2019). Moreover, the value of BAC and BCF also improved upon application of MEL; however, the values remained <1. The results also suggested the increased potential of Cu reclamation from soil in test plants sprayed with MEL. The maximum cleanup efficiency was observed in plants sprayed with 40 μM of MEL (Table 8). The metal uptake, translocation, and bioaccumulation by plants rely on metal concentration and their availability in soil and the morphological and physiological attributes of plants (Rajput et al., 2018). This may be attributed to improved growth and photosynthesis (Tables 2, 3) besides maintaining the cellular structure by reduction in ROS and LPO (Figures 2, 3), as well as maintaining cell viability (Figure 4) under higher Cu levels.

**FIGURE 5:**
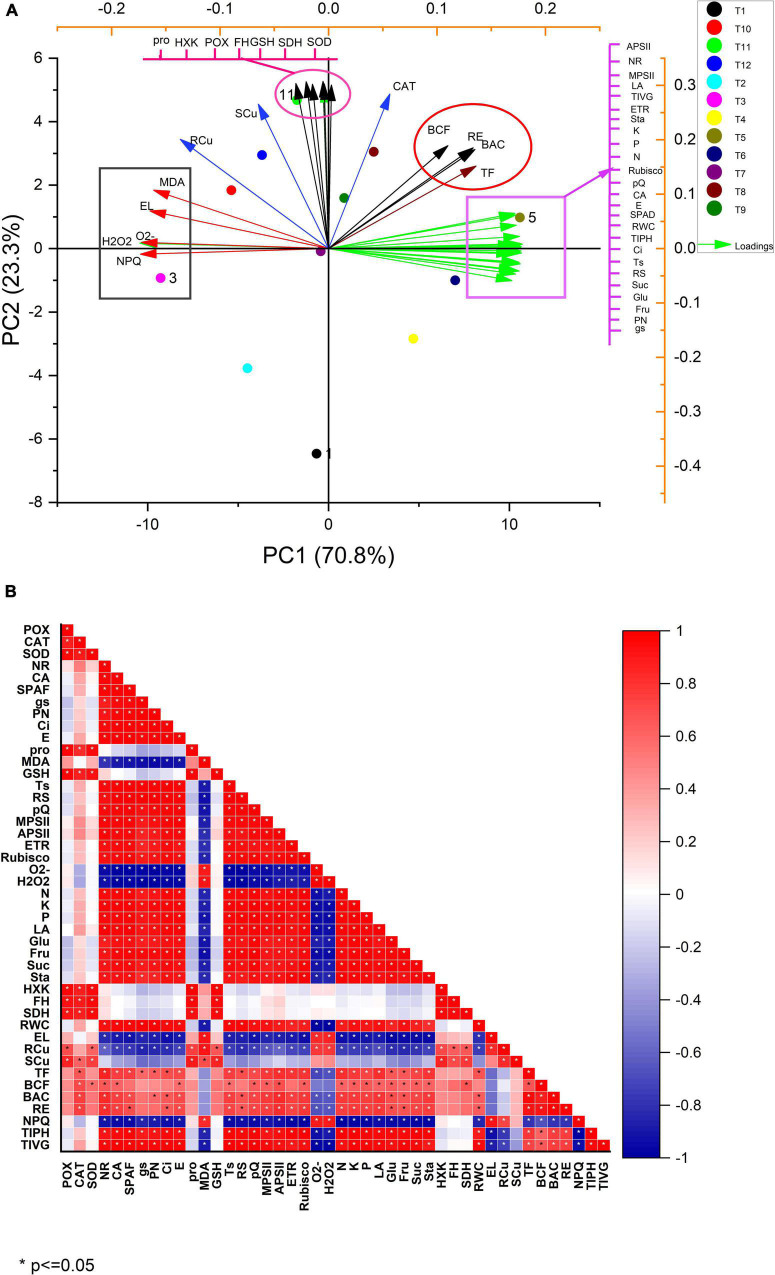
Correlation analysis by **(A)** Pearson correlation and **(B)** PCA analysis. T_1_, Control; T_2_, Cu (30 mg kg^–1^); T_3_, Cu (60 mg kg^–1^); T_4_, MEL (30 μM); T_5_, MEL (40 μM); T_6_, MEL (50 μM); T_7_, Cu (30 mg kg^–1^) + MEL (30 μM); T_8_, Cu (30 mg kg^–1^) + MEL (40 μM); T_9_, Cu (30 mg kg^–1^) + MEL (40 μM); T_10_, Cu (60 mg kg^–1^) + MEL (30 μM); T_11_, Cu (60 mg kg^–1^) + MEL (40 μM); T_12_, Cu (60 mg kg^–1^) + MEL (50 μM).

Pearson correlation was used to establish the relationship between the various parameters (Figure 5B). In addition, a principal component analysis (PCA) correlation between various parameters was investigated (Figure 5A). The score and loading plot of PCA showed a maximum (94.1%) variation among all the parameters studied, of which PC1 contributed a 70.8% variation whereas PC2 displayed a 23.3% variation. Moreover, most of the applied treatments were successfully displaced within the first two components that provided a clear indication that the application of MEL alone has a significant ameliorative effect on all the attributes relative to the Cu-stressed plants (Figure 5A). PC1 was positively influenced by variables having parameters APSII, NR, MPSII, LA, TIVG, ETR, K,P,N, rubisco activity, qP, CA, E, SPAD value, RWC, TIPH, Ci, TS, RS, Suc, Glu, Fru, PN, gs, TF, BAC, RE, and BCF), and PC2 with parameters containing NPQ, H_2_O_2_, O_2_^–^, EL, and MDA. A negative correlation was observed between PC1 and PC2. On the other hand, both PC1 and PC2, particularly PC2, contributed in the parameters like Pro, HXK, POX, FH, GSH, SDH, SOD, and CAT. In the case of the score plot, T_5_ (MEL 40 μM alone) had a maximum addition to PC1, where it represents a strong negative correlation to T_3_ (Cu 60 mg kg^–1^). The treatments (T_10_–T_12_) contributed maximally to PC2, whereas a small contribution was represented by treatments (T_7_ and T_9_). In contrast, T_8_ (Cu 30 mg kg^–1^ + MEL 40 μM) showed a positive relationship with PC1, thus confirming MEL’s potential stress-relieving role. In general, the amount of copper in the roots and shoots, and BCF were highly correlated with the amount of copper in the soil. On the other hand, various physio-biochemical, growth, and photosynthetic parameters, RWC, nutrient status, Cu translocation factor, BAC negatively contributed to Cu levels, but MEL spray had a favorable relationship. Furthermore, Cu stress elevated the ROS, MDA, and EL in plants, indicating a strong correlation between the two; however, negatively related to MEL spray. In addition, the application of MEL, in the presence or absence of Cu, enhanced the enzymatic and non-enzymatic antioxidants, proline levels, and related metabolic enzymes, all of which are positively correlated to both PC1 and PC2.

## Conclusion

The present experiment presented strong evidence that MEL remarkably increased the overall health of *Brassica juncea* plants by increasing plant tolerance and photosynthetic and chlorophyll fluorescence parameters, improving nutrient composition, and reducing the ROS, MDA, and EL content in plant tissues. MEL maintained the chloroplast and stomatal organization and consequently improved photosynthetic efficiency besides decreasing the apoptotic cell death in roots and leaves of the Cu-stressed plants. Additionally, the application of MEL proved effective in improving the Cu-reclamation ability from the soil, as evidenced by improved translocation of Cu from roots to aerial parts as well as increased BAC and BAF. The present study suggests that 40 μM of MEL spray could be an effective concentration in improving the stress resilience and phytoremediation potential of *B. juncea*.

## Data Availability Statement

The original contributions presented in this study are included in the article/Supplementary Material, further inquiries can be directed to the corresponding author.

## Author Contributions

AM: data collection with analysis and interpretation of the manuscript. SH: overall supervision throughout the analysis, preparation, and interpretation of the manuscript. PA: preparation and interpretation of the manuscript. All authors contributed to the article and approved the submitted version.

## Conflict of Interest

The authors declare that the research was conducted in the absence of any commercial or financial relationships that could be construed as a potential conflict of interest.

## Publisher’s Note

All claims expressed in this article are solely those of the authors and do not necessarily represent those of their affiliated organizations, or those of the publisher, the editors and the reviewers. Any product that may be evaluated in this article, or claim that may be made by its manufacturer, is not guaranteed or endorsed by the publisher.

## Abbreviations

APSII: actual PSII efficiency
BAC: bioaccumulation coefficient
BCF: bioconcentration factor
CAT: catalase
CA: carbonic anhydrase
Cu: copper
DDW: double distilled water
ETR: electron transport rate
EL: electrolyte leakage
FH: fumarase
Fru: fructose
Glu: glucose
GSH: glutathione
H_2_O_2_: hydrogen peroxide
OH: hydroxyl radical
HXK: hexokinase
*C*_*i*_: internal CO_2_ concentration
LA: leaf area
LPO: lipid peroxidase
MDA: malondialdehyde
MPSII: maximum PSII efficiency
MEL: melatonin
N: nitrogen
*P*_*N*_: net photosynthetic rate
NR: nitrate reductase
NBT: nitro-blue tetrazolium
NPQ: non-photochemical quenching
PGR: plant growth regulator
POX: peroxidase
qP: photochemical quenching
P: phosphorus
K: potassium
PCA: principal component analysis
Pro: proline
ROS: reactive oxygen species
RS: reducing sugar
RWC: relative water content
RE: removal efficiency
O_2_^–^: superoxide
SOD: superoxide dismutase
*g*_*s*_: stomatal conductance
Suc: sucrose
SDH: succinate dehydrogenase
TS: total sugar
*E*: transpiration rate
TF: translocation factor
TiPH: tolerance index expressed in terms of plant height
TiVG: tolerance index expressed in terms of vegetative growth.
