# Supplementary material for: Perspective of Melatonin-Mediated Stress Resilience and Cu Remediation Efficiency of Brassica juncea in Cu-Contaminated Soils
Source: Front Plant Sci. 2022 Jul 18;13:910714. doi: 10.3389/fpls.2022.910714 (PMC9340790; doi:10.3389/fpls.2022.910714)
Supplement: Supplementary file 1 [file Data_Sheet_1.docx]

| **Characteristics** | **Soil** |
| --- | --- |
| Texture | Sandy loam |
| pH | 7.79 ± 0.2 |
| NO_3_- (mg kg^−1^ soil) | 96.50 ± 0.015 |
| Phosphorous (mg kg^−1^ soil) | 81.20 ± 0.010 |
| Potassium (mg kg^−1^ soil) | 120.10 ± 1.06 |
| Calcium (mg kg^−1^ soil) | 18.35 ± 0.95 |
| Magnesium (mg kg^−1^ soil) | 30.80 ± 1.02 |
| Chloride (mg kg^−1^ soil) | 24.90 ± 1.22 |
| Sodium (mg kg^−1^ soil) | 11.10 ± 0.43 |
| Sulphate (mg kg^−1^ soil) | 16.72± 0.40 |
| Bicarbonate (mg kg^−1^ soil) | 17.95 ± 0.40 |
| Carbonate (mg kg^−1^ soil) | 75.61 ± 0.96 |
| Copper (mg kg^−1^ soil) | 18.0± 0.40 |

**F1: Physio-chemical properties of soil**
